# Historical gene flow constraints in a northeastern Atlantic fish: phylogeography of the ballan wrasse *Labrus bergylta* across its distribution range

**DOI:** 10.1098/rsos.160773

**Published:** 2017-02-15

**Authors:** Frederico Almada, Sara M. Francisco, Cristina S. Lima, Richard FitzGerald, Luca Mirimin, David Villegas-Ríos, Fran Saborido-Rey, Pedro Afonso, Telmo Morato, Sérgio Bexiga, Joana I. Robalo

**Affiliations:** 1 MARE—Marine and Environmental Sciences Centre, ISPA Instituto Universitário, Rua Jardim do Tabaco 34, 1149-041 Lisboa, Portugal; 2 Carna Research Station, Ryan Institute, National University of Ireland, Galway, Republic of Ireland; 3 Marine and Freshwater Research Centre, Galway-Mayo Institute of Technology, Dublin Road, Galway, Republic of Ireland; 4 Institute of Marine Research (IMR), Flødevigen Marine Research Station, 4817 His, Norway; 5 Institute of Marine Research (IIM-CSIC), Vigo, Spain; 6 MARE—Marine and Environmental Sciences Centre, Universidade dos Açores, 9901-862 Horta, Portugal; 7 Departamento de Oceanografia e Pescas da Universidade dos Açores, IMAR—Institute of Marine Research, 9901-862 Horta, Portugal

**Keywords:** population structure, Labridae, cleaner fish, glacial refugia, Azorean distinctiveness, incipient speciation

## Abstract

The distribution and demographic patterns of marine organisms in the north Atlantic were largely shaped by climatic changes during the Pleistocene, when recurrent glacial maxima forced them to move south or to survive in northern peri-glacial refugia. These patterns were also influenced by biological and ecological factors intrinsic to each species, namely their dispersion ability. The ballan wrasse (*Labrus bergylta*), the largest labrid fish along Europe's continental margins, is a target for fisheries and aquaculture industry. The phylogeographic pattern, population structure, potential glacial refugia and recolonization routes for this species were assessed across its full distribution range, using mitochondrial and nuclear markers. The existence of a marked population structure can reflect both recolonization from three distinct glacial refugia and current and past oceanographic circulation patterns. Although isolated in present times, shared haplotypes between continental and Azores populations and historical exchange of migrants in both directions point to a common origin of *L. bergylta*. This situation is likely to be maintained and/or accentuated by current circulation patterns in the north Atlantic, and may lead to incipient speciation in the already distinct Azorean population. Future monitoring of this species is crucial to evaluate how this species is coping with current environmental changes.

## 1. Background

The northeastern Atlantic experienced considerable climatic changes during the Pleistocene when glacial cycles acted as a major driver shaping the structure of marine communities [1]. With the glaciation of the North Sea and the loss of the warm temperate regime along the west European continental margins [1–3], marine populations were forced to move towards southernmost regions or to survive in northern peri-glacial refugia [4]. During glacial maxima the polar front extended far south and reached the north of the Bay of Biscay [3] and possibly the western Iberian coast [2,5]. Despite the drastic changes in sea surface temperature [6], several glacial refugia have been evidenced, including areas around northern Norway, the Faeroes and Iceland, the Hürd Deep, southwest Ireland and southwest Britain, southwest Continental Europe and the Mediterranean Sea [4]. The rising temperatures and resulting regression of the polar front during interglacial periods would then allow the recolonization of northern regions [7].

These long-term environmental changes led to the emergence of distinct distribution patterns and, at the same time, to similarities between the geographical ranges of species that share similar environmental tolerance ranges [8]. Within this framework, many coastal species are thought to have experienced expansions and contractions of their geographical ranges associated with demographic changes, reflecting their biogeographic origins and environmental tolerances. As such, it could be expected that warm temperate and tropical species may have only survived in the warmer Mediterranean or along the West African coast, while cold tolerant species may have also survived in northern refugia [9]. In addition, there are multiple factors involved in the changing geographical distribution of populations and species, some of them related to other environmental variables, species-specific characters such as life-history traits and stochastic effects.

It should also be expected that distinct species coped differently with the above-referred constraints. Even closely related sympatric species with similar environmental tolerances may present distinct genetic structures and demographic history patterns (for a review see [10–12]). This should explain why we can find in the northeast Atlantic fish species showing: (i) panmictic populations without latitudinal differences in genetic diversity (e.g. *Lipophrys pholis* [13]); (ii) significant population structure but still with similar levels of genetic diversity throughout the entire species range (e.g. *Taurulus bubalis* [14]); or (iii) sharp decline of genetic diversity from southern (west European) to northern (Scandinavian) populations (e.g. *Pomatoschistus microps* [15]; *Symphodus melops* [16]). Similarly, studies have indicated that some species may have persisted in northern regions even during glacial maxima (e.g. *Pholis gunnellus* [17]), while other only subsisted near the North Sea [14,15] or within the Mediterranean (e.g. *Chromis chromis* [18]; *Sprattus sprattus* [19]).

The ballan wrasse, *Labrus bergylta* Ascanius, 1767, is the largest labrid fish in continental waters of the northeastern Atlantic [20], presently ranging from southern Norway to the west coast of Morocco and the Macaronesian archipelagos [21]. It is a sedentary, obligate protogynous hermaphrodite species whereby dominant territorial males derive from sex-changing mature females, which typically live in harems [22]. Adhesive eggs are laid in rocky substrates and larvae are released to the plankton [23].

It has attracted considerable attention due to three main reasons: (i) it is a target for commercial [22] and recreational fisheries [24], (ii) it is currently used as a cleaner species in northern Europe salmonid fish farms as an alternative to chemical treatments [25,26], and (iii) its phenotypic plasticity and life-history variation have raised questions about its taxonomic status and the possibility that one or more cryptic species could exist.

In recent years, several consensual and quite complete phylogenies have been published on both the tribe Labrini (where the genus *Labrus* is included) [27] and on the family Labridae [28]. At the same time, the systematic classification of the northeastern Atlantic and Mediterranean Labridae had been revised due to the recent split of some taxa and the high intraspecific polymorphism, particularly in the genera *Labrus* and *Symphodus* ([27,29] and references therein). Hanel *et al.* [27] reported high intraspecific genetic divergence in *L. bergylta* and its systematic status has raised additional interest because [30] (and references therein) showed differences in life-history traits of two main morphotypes (plain and spotted body colour patterns). Genetic differences between morphotypes were reported in [31] but only with microsatellites in one restricted region. Recently, [32] found no differences between morphotypes with genetic markers widely used in fish phylogeography.

A phylogeographic study of the ballan wrasse based on the control region (CR) of mitochondrial DNA has recently been published in [33]. Focusing in the northern distribution range of the species, around the British Isles and southern Norway, this study showed reduced levels of mitochondrial genetic diversity towards northern latitudes, and the presence of two divergent clades showing evidence of population expansion.

This study extends the previous study in [33] and our potential knowledge of the species phylogeography and genetic structure by: (i) covering the species geographical distribution and (ii) analysing a nuclear fragment (the first intron of the S7 ribosomal protein gene) in addition to the mitochondrial CR fragment. The present genetic assessment will aid conservation and management of the species by providing essential information: (i) on potential effects associated with individual translocations or the exploitation of wild cleaner fish in salmonid fish farms in the North Sea and (ii) for stock assessment studies of local fisheries in the central northeastern Atlantic. To date this is the most comprehensive study describing the genetic structure, the signatures of expansion/contraction events and identifying potential Pleistocene refugia of the ballan wrasse along its distributional range.

## 2. Material and methods

### 2.1. Sampling

**Figure 1.**
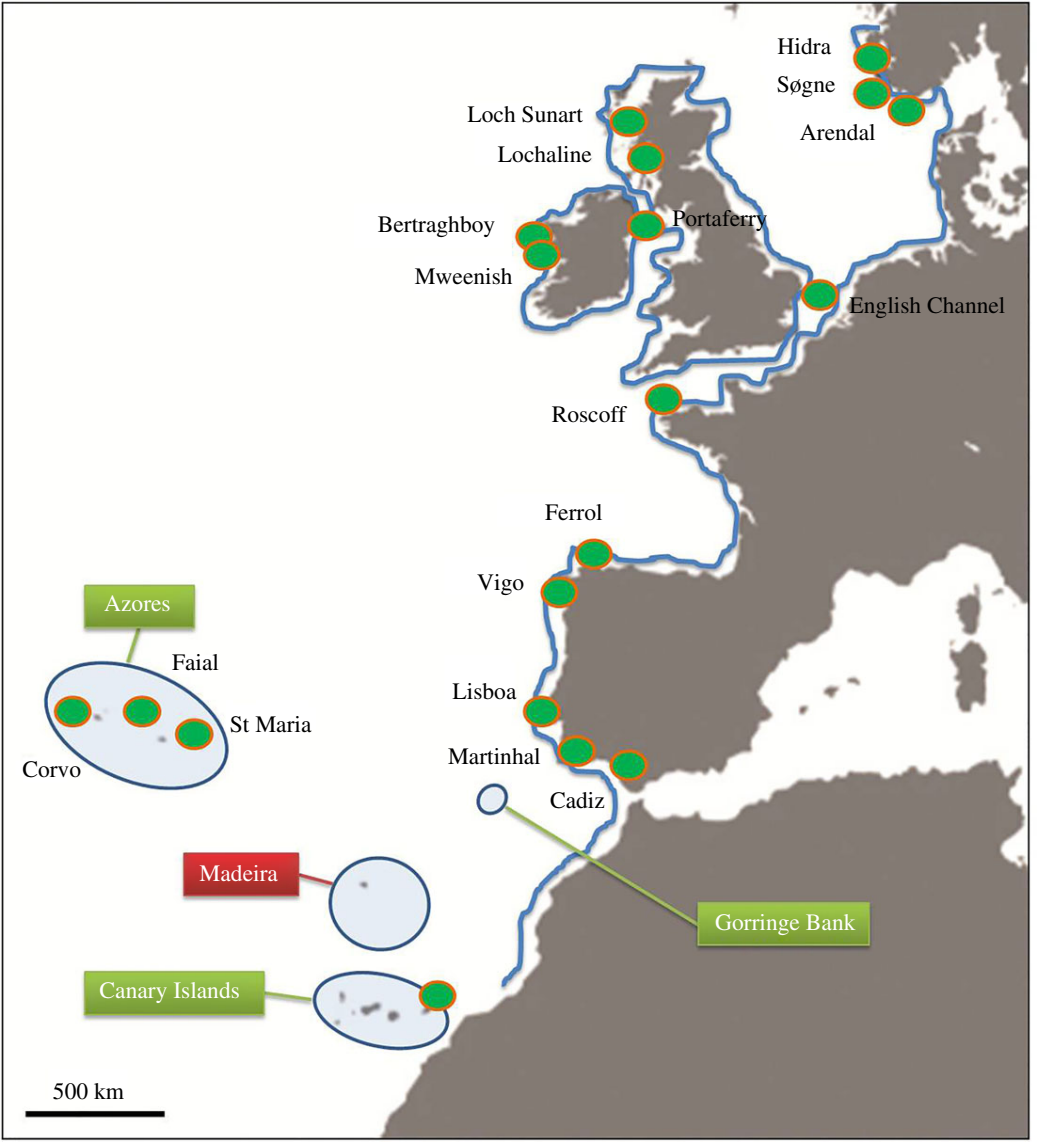
Map of sampling locations for *Labrus bergylta*.

Specimens of *L. bergylta* were obtained from 14 locations along its distributional range in the northeastern Atlantic and North Sea (figure 1 and table 1). These sites included: Corvo, CO and Santa Maria, SM in the Atlantic archipelago of the Azores; Lisbon, LI in western Portugal; Vigo, VI and Ferrol, FE in northern Spain (Galicia); Roscoff, RO in France; Portaferry, PO in Northern Ireland; Mweenish, ME and Bertraghboy Bay, BB in western Ireland; Lochaline, LO and Loch Sunart, LS in western Scotland; Arendal, AR, Hidra, HI and Sogne, SO in southern Norway.

**Table 1.** Diversity measures for the collecting sites and population groups of *Labrus bergylta* for CR and S7: number of sequences (*N*), number of haplotypes (Nh), private haplotypes (Ph), haplotype diversity (*h*), nucleotide diversity (*π*) and mean number of pairwise differences (PD).

			CR						S7					
location	label	coordinates	*N*	Nh	Ph (%)	*h*	*π*	PD	*N*	Nh	Ph (%)	*h*	*π*	PD
Arendal	AR	58°25′ N, 08°45′ E	17	7	57	0.721	0.018	6.015	—	—	—	—	—	—
Hidra	HI	58°13′ N, 06°31′ E	49	12	42	0.605	0.020	6.624	44	10	10	0.839	0.003	1.398
Sogne	SO	58°04′ N, 07°48′ E	50	10	40	0.481	0.009	3.109	46	11	9	0.847	0.003	1.523
*Norway*			116	19	74	0.569	0.015	5.091						
Loch Sunart	LS	56°40′ N, 05°56′ W	24	15	27	0.909	0.028	9.398	20	9	0	0.879	0.003	1.658
Lochaline	LO	56°31′ N, 05°46′ W	26	17	24	0.938	0.025	8.335	40	10	20	0.869	0.003	1.613
Portaferry	PO	54°23′ N, 05°33′ W	14	13	62	0.989	0.027	9.044	24	6	17	0.786	0.003	1.406
Bertraghboy	BB	53°19′ N, 09°51′ W	44	22	64	0.970	0.029	9.616	—	—	—	—	—	—
Mweenish	ME	53°17′ N, 09°49′ W	72	39	54	0.964	0.026	8.533	44	12	17	0.849	0.003	1.571
Roscoff	RO	48°43′ N, 03°58′ W	27	22	68	0.977	0.023	7.547	46	12	17	0.850	0.003	1.543
Ferrol	FE	43°27′ N, 08°17′ W	20	14	36	0.953	0.009	2.858	36	7	0	0.841	0.002	1.367
Vigo	VI	42°13′ N, 08°46′ W	21	17	47	0.976	0.017	5.814	26	11	18	0.901	0.003	1.692
Lisbon	LI	38°42′ N, 09°24′ W	34	22	59	0.955	0.019	6.241	68	14	21	0.810	0.002	1.302
*Europe*			282	120	96	0.964	0.024	7.857	394	27	96	0.856	0.003	1.533
Santa Maria	SM	36°57′ N, 25°06′ W	15	13	92	0.971	0.013	4.362	24	5	20	0.696	0.002	0.880
Corvo	CO	39°41′ N, 31°05′ W	15	12	92	0.971	0.016	5.343	26	5	40	1.062	0.002	1.062
*Azores*			30	24	100	0.982	0.015	4.880	50	7	86	0.696	0.002	0.971
all			428	158	—	0.946	0.037	12.198	444	33	—	0.862	0.003	1.583

All sampling and handling of fish were performed by experienced personnel in accordance with relevant legislation in each country (see the Ethics section for detailed information). A field campaign to Madeira in 2014 provided no fish samples or even sightings despite this species being listed to the region [34]. In all cases, a small piece of fin tissue was clipped and preserved in 96% ethanol.

### 2.2. DNA extraction, amplification and sequencing

Total genomic DNA was extracted with the REDExtract-N-Amp Kit (Sigma-Aldrich) following the manufacturer's instructions. The mitochondrial CR and the first intron of the nuclear S7 ribosomal protein gene (S7) were amplified using L-pro1 and H-DL1 [35], and S7RPEX1F and S7RPEX2R [36], respectively (see electronic supplementary material, appendix I for details).

Sequences were edited with Codon Code Aligner (Codon Code Corporation) and aligned with Clustal X 2.1 [37]. Whenever possible, both strands of the same specimen were recovered for S7 following the approach of [38]. Sequences obtained were deposited in GenBank (accession nos. KU751889-KU752182). Additional sequences available in GenBank were obtained from [32,33] (electronic supplementary material, table S1 provides the original reference for each individual sequence).

### 2.3. DNA analyses

The appropriate model of sequence evolution for each fragment was determined using the jModeltest program [39,40], applying the Akaike information criterion [41]. Haplotype networks were built with the software TCS 1.21 [42] using the parsimony method in [43]. For these analyses, some additional sequences from Faial, FA (Azores); Canary Islands, CI; Martinhal, MA and Cádiz, CA (south Iberia); and English Channel, EC were added in order to extend the sampling area (electronic supplementary material, table S1).

The ARLEQUIN software package v. 3.5 [44] was used to estimate genetic diversity within each sample, to evaluate potential population differentiation and to perform neutrality tests. The same software was used to perform analyses of molecular variance (AMOVA) [45], to compute pairwise *F*_ST_ estimates and corrected pairwise differences between populations. In the case of the S7 gene fragment, the analyses were also run in ARLEQUIN after allowing the program to reconstruct the haplotypes using the ELB algorithm [46]. The number of migrants was calculated for each population pair using the same software. The correlation between geographical distance and *F*_ST_ was computed with the Mantel test [47,48] (also in ARLEQUIN with 10 000 permutations; geographical distances measured along the shore line).

The spatial analysis of molecular variance (SAMOVA 1.0) [49] was used to identify groups of sampling locations that are geographically and genetically homogeneous and maximally differentiated from each other. The most likely number of groups was identified by running SAMOVA with 2 to 13 groups and choosing the partition scheme with the highest *F*_CT_ value. The sequences of the locations included in each of the groups that maximized *F*_CT_ were pooled together. Mismatch analysis [50,51], Fu's *F*s [52] and Tajima's *D* [53] tests were performed in ARLEQUIN to test for possible bottlenecks and population expansion in each group. For the mismatch analyses parameters *τ*, *θ*_0_, *θ*_1_ and *M* and their confidence intervals were obtained by a parametric bootstrap approach using 10 000 replicates. Effective population sizes were calculated before (*N*_0_) and after (*N*_1_) a sudden expansion event, the effective population size prior to a spatial expansion event (*N*), the time since the expansion (*t*, in years), and the migration rate (*m*), using the equations *τ* = 2*μτ* and *θ* = 2N*μ*.

A Markov chain Monte Carlo (MCMC) approach taking into account phylogenetic relationships among haplotypes as implemented in LAMARC 2.1.9 [54] was used to estimate effective population size (Nef), the exponential growth parameter (*g*) and the migration rates among adjacent groups of populations, using 10 runs of 12 short chains of 1000 steps and five long chains of 50 000 steps, with a burn-in of 10 000 steps. In order to compute estimates of effective population size, their changes with time and the age of populations, the following mutation rates were used: 5% for CR [55] and 0.23% for S7 [56]. MtDNA CR mutation rates in fish are widely variable (e.g. 2.2–4.5%/MY between lineages for East African cichlids [57]; 15–20%/MY for Indo-Pacific sardines [58]). In the absence of a clock calibration for the CR of *L. bergylta*, we address the uncertainty by tentatively assuming a within lineage mutation rate of 5%/MY, which is within the range of values found for other fish species

Past population demography of *L. bergylta* was inferred using the linear Bayesian skyline plot (BSP) model [59] as implemented in BEAST v. 1.7 [60], employing the Bayesian MCMC coalescent method and a strict clock. The Bayesian distribution was generated using results from five independent runs of 150 million MCMC steps obtaining effective samples sizes (ESS) of parameter estimates of over 200, with a burn-in of 10%. The time to most recent common ancestor (tMRCA) and the median and corresponding credibility intervals of the BSP were depicted using Tracer v. 1.6 [61].

## 3. Results

The CR dataset consisted of a total of 333 bp fragment after alignment (433 sequences) and yielded 160 distinct haplotypes with 93 polymorphic sites (94 transitions, 12 transversions and six indels). For the S7, a fragment of 537 bp was analysed, with 39 haplotypes obtained for the 478 sequences (corresponding to 239 individuals). Differences between haplotypes included 12 transitions, six transversions and no indels, in 18 polymorphic sites.

**Figure 2.**
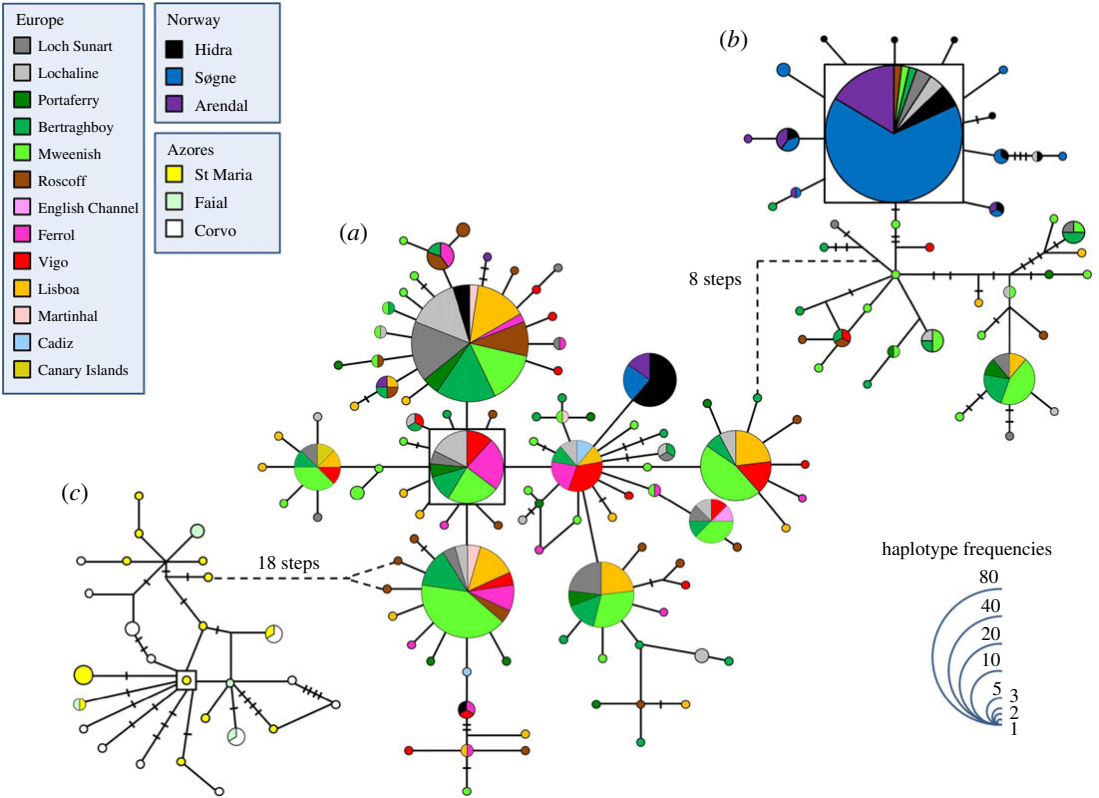
(*a–c*) Haplotype network for the CR of *Labrus bergylta*. The haplotype with the highest out group probability is displayed as a square, other haplotypes as circles. The area of the circles is proportional to each haplotype frequency. Colours refer to the region in which haplotypes were found. In the case where haplotypes are shared among regions, shading is proportional to the frequency of the haplotype in each region.

**Figure 3.**
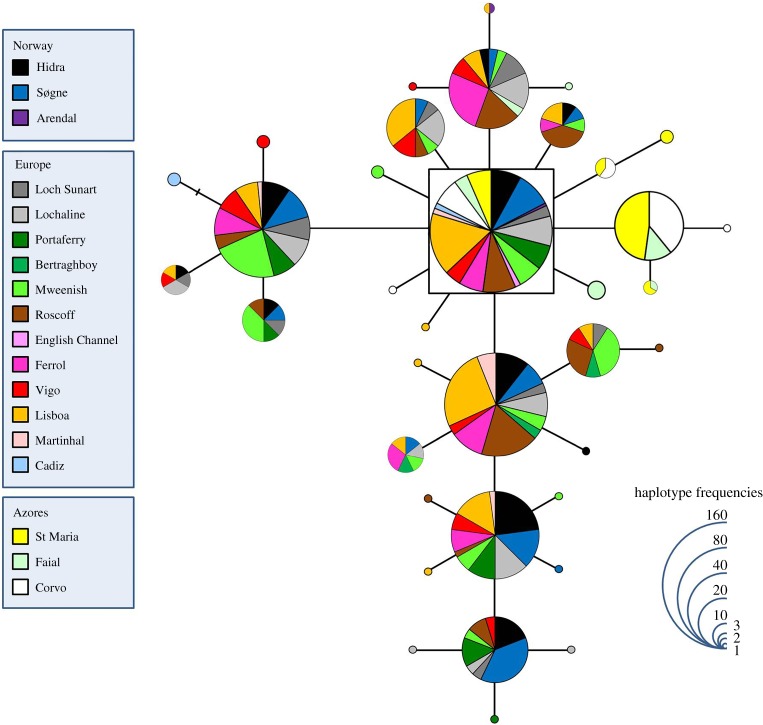
Haplotype network for the S7 of *Labrus bergylta*. The haplotype with the highest out group probability is displayed as a square, other haplotypes as circles. The area of the circles is proportional to each haplotype frequency. Colours refer to the region in which haplotypes were found. In the case where haplotypes are shared among regions, shading is proportional to the frequency of the haplotype in each region.

The haplotype network for the CR of *L. bergylta* revealed three distinct groups (figure 2). The first group (sub-network (*a*) in figure 2) included most sequences from the Atlantic European shores and some Norwegian samples, showing star-like patterns with haplotypes shared between several sampling locations. The inferred ancestral haplotype included 17 individuals from Spain, Ireland and Scotland (outgroup weight 0.096). The second sub-network grouped ((*b*) in figure 2) together the majority of the Norwegian and the remaining Atlantic European sequences. The ancestral haplotype inferred for this sub-network included 82 fish from Norway, Ireland, Scotland and France (outgroup weight 0.238). Interestingly, the only sample caught in the Canary Islands shared its haplotype with fish from Ireland, Scotland, northern Spain and western Portugal. The third sub-network ((*c*) in figure 2) includes only fish from the Azores (outgroup weight 0.109). The haplotype network obtained for the S7 of the ballan wrasse is less deep and diverse, and the geographical pattern obtained is not as obvious (figure 3). The inferred ancestral, dominant and in the centre of several star-like patterns, includes 138 fish from Norway, Ireland, Scotland, the English Channel, France, Spain and Portugal (mainland and Azores) (outgroup weight 0.104).

Table 1 showed the genetic diversity indices per sampling site. For the CR, haplotype diversity indices were higher for the Atlantic locations, comparing to Norwegian ones. Azorean locations presented a much higher percentage of private haplotypes when compared with all other sites. Concerning the S7, the genetic indices showed homogeneity along sites.

AMOVA analyses revealed genetic structure along the distribution area of *L. bergylta* for both genetic markers (CR: *F*_ST_ = 0.462, *p* < 0.001; S7: *F*_ST_ = 0.107, *p* < 0.001). Genetic differentiation and gene flow between sampling locations are shown in table 2 and corrected pairwise differences in electronic supplementary material, table S2. For the CR, significant *F*_ST_ and corrected pairwise differences were found between locations from Norway, Azores and the rest of European sites, confirming the existence of three populations. Significant differences were also found between FE and some other adjacent locations; however, migration among these locations was consistently higher than the threshold limit of 1. The pattern of differentiation was not as straightforward for the S7. Significant *F*_ST_ and corrected pairwise differences were found between Azorean and all other sites. These sites revealed no evident pattern, with significant differences being found between some of them. Considering the number of migrants (Nm), isolation was only found between the Azores and the remaining locations.

**Table 2.** Gene flow among collecting sites of *Labrus bergylta* represented by *F*_ST_ (below diagonal) and Nm (number of migrants; above diagonal). Significant values of probability *p* are shown in italics.

CR	AR	HI	SO	LS	LO	PO	BB	ME	RO	FE	VI	LI	SM	CO
AR		inf	60.345	0.633	0.595	0.619	0.727	0.573	0.440	0.205	0.354	0.348	0.130	0.145
HI	−0.026		9.562	0.699	0.684	0.701	0.799	0.654	0.509	0.305	0.447	0.422	0.154	0.164
SO	0.008	*0*.*050*		0.304	0.293	0.370	0.387	0.254	0.229	0.117	0.183	0.195	0.078	0.085
LS	*0*.*442*	*0*.*417*	*0*.*622*		inf	inf	inf	inf	inf	5.787	38.305	31.941	0.195	0.212
LO	*0*.*457*	*0*.*422*	*0*.*630*	−0.032		inf	inf	inf	inf	6.311	184.129	32.578	0.180	0.196
PO	*0*.*466*	*0*.*433*	*0*.*663*	−0.037	−0.039		inf	inf	inf	5.300	inf	98.994	0.174	0.193
BB	*0*.*408*	*0*.*385*	*0*.*563*	−0.020	−0.018	−0.036		inf	32.978	5.172	29.067	15.283	0.219	0.234
ME	*0*.*447*	*0*.*416*	*0*.*575*	−0.013	−0.014	−0.033	−0.011		46.576	6.718	73.917	25.630	0.203	0.214
RO	*0*.*532*	*0*.*496*	*0*.*686*	−0.005	−0.003	−0.011	0.015	0.011		25.520	inf	inf	0.166	0.180
FE	*0*.*709*	*0*.*621*	*0*.*810*	*0*.*080*	*0*.*073*	*0*.*086*	*0*.*088*	*0*.*069*	0.019		26.002	505.969	0.081	0.094
VI	*0*.*586*	*0*.*528*	*0*.*732*	0.013	0.003	−0.006	0.017	0.007	−0.005	0.019		inf	0.125	0.140
LI	*0*.*590*	*0*.*543*	*0*.*719*	0.015	0.015	0.005	0.032	0.019	−0.010	0.001	−0.013		0.139	0.150
SM	*0*.*793*	*0*.*764*	*0*.*865*	*0*.*720*	*0*.*735*	*0*.*742*	*0*.*695*	*0*.*712*	*0*.*751*	*0*.*860*	*0*.*799*	*0*.*783*		44.69298
CO	*0*.*775*	*0*.*753*	*0*.*855*	*0*.*702*	*0*.*718*	*0*.*721*	*0*.*682*	*0*.*701*	*0*.*735*	*0*.*842*	*0*.*781*	*0*.*769*	0.011	

S7	HI	SO	LS	LO	PO	ME	RO	FE	VI	LI	SM	CO		
HI		inf	6.893	38.323	inf	24.919	6.286	8.191	10.416	6.540	0.773	0.834		
SO	−0.014		8.104	30.246	inf	36.058	6.526	7.881	9.747	5.638	0.832	0.905		
LS	*0*.*068*	*0*.*058*		inf	9.797	1653.034	11.143	inf	inf	10.972	1.315	1.506		
LO	0.013	0.016	−0.009		22.087	312.661	12.842	53.152	inf	23.044	1.133	1.284		
PO	−0.003	−0.015	0.049	0.022		inf	3.958	5.402	13.078	3.684	0.744	0.781		
ME	0.020	0.014	0.000	0.002	−0.005		7.616	12.118	355.708	7.606	1.109	1.257		
RO	*0*.*074*	*0*.*071*	0.043	*0*.*037*	*0*.*112*	*0*.*062*		153.494	8.173	152.166	1.311	1.540		
FE	*0*.*058*	*0*.*060*	−0.002	0.009	*0*.*085*	*0*.*040*	0.003		69.923	80.944	1.219	1.415		
VI	*0*.*046*	*0*.*049*	−0.037	−0.014	0.037	0.001	*0*.*058*	0.007		10.130	1.178	1.327		
LI	*0*.*071*	*0*.*081*	*0*.*044*	0.021	*0*.*120*	*0*.*062*	0.003	0.006	0.047		1.261	1.510		
SM	*0*.*393*	*0*.*375*	*0*.*275*	*0*.*306*	*0*.*402*	*0*.*311*	*0*.*276*	*0*.*291*	*0*.*298*	*0*.*284*		inf		
CO	*0*.*375*	*0*.*356*	*0*.*249*	*0*.*280*	*0*.*390*	*0*.*285*	*0*.*245*	*0*.*261*	*0*.*274*	*0*.*249*	−0.007			

SAMOVA results for the CR dataset yielded a maximized *F*_CT_ (0.623, *p *= 0.009) for two groups, the two locations from the Azores (CO and SM) versus the 12 locations belonging to other European shores (AR, HI, SO, LS, LO, PO, BB, ME, RO, FE, VI and LI). The three gene pools structured test (Azores versus Norway versus all remaining sites in Continental Europe) was also significant (*F*_CT_ = 0.610, *p *< 0.001). Concerning the S7, the SAMOVA analysis revealed a maximized *F*_CT_ (0.268, *p* = 0.016) for two gene pools: Azores versus all remaining sites.

Isolation by distance was found for both the CR and the S7 of the ballan wrasse, as the Mantel test yielded significant correlations between genetic and geographical distances (*r* = 0.738, *p* < 0.001 and *r* = 0.573, *p* < 0.001, respectively). Taking together the results from the *F*_ST_, Nm and SAMOVA, the following groups were considered for further demographic and phylogeographic analyses: three populations for the CR—Azores (CO and SM) versus Atlantic (LS, LO, PO, BB, ME, RO, FE, VI and LI) versus Norway (AR, HI and SO); and two populations for the S7—Azores (CO and SM) versus Continental Europe (HI, SO, LS, LO, PO, ME, RO, FE, VI and LI). These two schemes ensured highly significant differences (*p* < 0.001) among groups of populations: CR (*F*_ST_ Azores-Atlantic = 0.718, *F*_ST_ Azores-Norway = 0.801 and *F*_ST_ Atlantic-Norway = 0.519) and S7 (*F*_ST_ Azores-Continental Europe = 0.718).

Neutrality tests yielded negative values for both markers (table 3) with the exception of the Azorean population for the S7. However, significant values suggesting demographic expansion were only present for the *F*_S_ of Azores and Atlantic (CR) and Continental Europe (S7).

**Table 3.** Demographic parameters of *Labrus bergylta* based on CR and S7. Significant values of probability *p* are shown with an asterisk. Neutrality tests: *F*s (Fu's), *D* (Tajima's). Mismatch distributions: *t* (time in years, 95% CI in parenthesis), *N*_0_ (female effective population size before the expansion), *N*_1_ (female effective population size after the expansion), SSD (sum of square deviation) and Hri (Harpending's Raggedness index). Estimates of population parameters with LAMARC: *θ* (theta), *N*_f_ (female effective population size), *G* (growth rate) and *N*_1%_ (age of population, accessed as the age at which *N*_f_ drops below 1%). Estimates of tMRCA (time to most recent common ancestor) with BEAST. n.a., not applicable. nc, no convergence.

	CR			S7	
	Azores	Atlantic	Norway	Azores	Continental Europe
neutrality tests					
* F*_S_	−17.880*	−24.339*	−0.939	−2.083	−17.224*
* D*	−1.284	−1.068	−0.306	0.199	−0.368
mismatch distribution					
* demographic expansion*					
* t* (95% CI) (ky)	154 (114–185)	46 (16–261)	—	—	—
* N*_0_	106	130 378	—	—	—
* N*_1_	3 002 972 973	3 002 972 973	—	—	—
* *SSD	0.008	0.017	0.406*	0.012	0.011*
* *Hri	0.029	0.011	0.112	0.125*	0.102*
* spatial expansion*					
* t* (95% CI) (ky)	154 (97–180)	471 (28–898)	519 (10–868)	—	—
* N*	22	260 213	27 244	—	—
* *Nm	3 002 972 973	4848	12 426	—	—
* *SSD	0.008	0.026	0.027	0.012*	0.011*
* *Hri	0.029	0.011	0.112	0.125*	0.102*
LAMARC					
* θ* (95% CI)	0.196 (0.069–0.749)	0.068 (0.033–0.181)	0.003 (0.001–0.009)	0.005 (0.001–0.036)	0.006 (0.002–0.041)
* N*_f_ (95% CI)	1 960 888 (692 093–7 498 888)	678 830 (332 300–1,812,773)	30 758 (11 270–93 085)	1,082,120 (260 181–7,854,130)	1 369 457 (398 533–8,842,717)
* G* (95% CI)	361 (149–750)	87 (−11–306)	−154 (−757–314)	1230 (−1323–8023)	1041 (−549–6,940)
* N*_1%_ (95% CI) (ky)	255 (123–619)	1060 (300–n.a.)	n.a. (293–n.a.)	1628 (250–n.a.)	1923 (288–n.a.)
BEAST					
* t*_MRCA_ (95% CI) (ky)	220 (49–2230)	nc	548 (113–4616)	0 (0–0)	nc

The analyses of the mismatch distributions for the CR of *L. bergylta* were compatible with the models of sudden and spatial expansion for the Azores and all remaining Atlantic populations, except for the sudden demographic expansion in the case of Norway (table 3). Visual inspection of the mismatch graphics reveals clear differences among the three populations: the Azorean population presents a narrower width in comparison with the other two populations, revealing a strong peak at five differences; the Atlantic population revealed a stronger peak at four differences, while Norway presented a peak at 0 differences (electronic supplementary material, figure S1). The average estimated time for the demographic expansion yielded 154 thousand years (ky) for the Azores and 46 ky for the Atlantic. Considering the average spatial expansion, it was estimated to have occurred in the Late Pleistocene for the Azores (154 kya) and in the Middle Pleistocene for the Atlantic (471 kya) and Norway (519 kya). The S7 dataset, on the other hand, did not conform to neither the demographic nor the spatial model, for both populations.

For the CR dataset of the ballan wrasse, higher female effective population size was found for the population of Azores, and the Norwegian group presented the lowest value for this parameter. The same pattern was found for the growth rates, with the Scandinavian population presenting a negative growth rate. For the S7, the yielded values were similar for the two analysed groups (table 3). BSP runs failed to converge for the CR of the Atlantic group and for the S7 of Continental Europe. Estimates of tMRCA were consistent with estimates from previous analyses.

For the CR of *L. bergylta*, it was not possible to estimate migration rates between the Azorean group and the rest, as the connectivity assumption of LAMARC was not met. Migration rate was higher from Norway to the Atlantic (20.564) than in the opposite direction (0.014). Considering the S7 dataset, the migration rate was higher from the Azores into the European group (390.521) versus 106.857 in the opposite direction.

## 4. Discussion

The results presented in this study highlight four main features concerning the phylogeography of the ballan wrasse. First, the northward decrease in genetic diversity along the species' distributional range. Second, the existence of genetic structure along the sampled area. Third, the distinctiveness of the Azorean population. Finally, the demographic and spatial expansion of most of the species' populations. Taken together, these findings agree with and expand those found in [33] for the North Sea.

### 4.1. Genetic diversity and population structure

In general, the pattern of haplotype diversity found for the ballan wrasse follows that reported for other fishes [15,16], with higher indices in the central north Atlantic and lower in the northern locations (CR dataset). The Azorean locations clearly stood out, with higher percentages of private haplotypes. This fact supports the distinctiveness of the population from this isolated archipelago in the central northern Atlantic. Similar results were described for other fish species like *Coryphoblennius galerita* [62] and *L. pholis* [63], calling attention to the genetic distinctiveness and importance of the Azorean fish populations.

Are these differences related to life-history traits such as the pelagic larval duration (PLD)? The correlation between the PLD and *F*_ST_ estimates has been found to be very strong in recent modelling approaches [64]. However, there is an ongoing debate on this topic, as other studies (e.g. [65]) refute the conventional use of PLD as a good predictor for the magnitude of gene flow. Several studies reported the absence of genetic structure for species with relatively long PLD, such as the rocky intertidal *L. pholis* [13] or the highly migratory *Thunnus thynnus* [66]. Although somehow expected, this is not a consensual pattern for species with such a life-history trait. In fact, population structure has also been recorded for a variety of fishes [14,62]. This also seems to be the case of the ballan wrasse, which has a relatively long PLD of 37–49 days [67] but revealed genetic structure along the sampled area, especially when considering the CR dataset. The locations from Norway, Azores and the rest of European sites were considered to be significantly different. Although the results from the slow evolving S7 gene do not show such a clear cut, Azores keeps its singularity, being isolated (with no migration) from the remaining European locations (average Nm of 0.160 between Azorean locations and the remaining ones). Similar findings related to the peripheral position of Azorean haplotypes were also reported for several other species [62,68].

Are these differences related to ocean current patterns in the northeastern Atlantic? The North Atlantic circulation pattern can at least partially explain the isolation of the Azorean population as it prevents gene flow between European and Azorean locations. This isolation is mainly related to the Azores recent Miocene origin (1–8 My [69–71]), its distance from the closest continental shore (approx. 1300 km from mainland Europe), and the fact that predominant sea surface currents cross the North Atlantic in an eastward direction. Main current patterns may show regional variations that can be detected at a seasonal scale [72], yearly scale from decades to thousand years [73], or may occasionally generate considerable mesoscale variability (e.g. according to [74] strong eddies promote northwestward transport from Africa or Madeira towards the Azores).

### 4.2. Complexity and depth of the genealogies

All the analyses reveal expansion events and population ages clearly older than the Last Glacial Maximum (LGM, 21–18 kya) [75] with lineages coalescing in Late and Middle Pleistocene. Similar findings were reported for other fish in northeastern Atlantic (e.g. [62]). The close relative *S. melops*, on the other hand, showed a different pattern with exclusive haplotypes for the Scandinavian population and an expansion event much younger than the LGM [16].

Migration rate was higher from Norway to the Atlantic than in the opposite direction (CR), unlike what was found for other species (e.g. [14,16]). Considering the S7 dataset, the migration rate was higher from the Azores into the European group compared with the opposite direction. This pattern is uncommon and needs further evaluation. Nevertheless, this historical migration was previously advanced in [76] in a model of blenniid speciation in the northeastern Atlantic. According to this author, fish from European mainland could occasionally be transported to the Atlantic islands and survive glacial maxima, potentially evolving to become incipient new species. The Azores would thus act as potential speciation centre, exporting them back to European shores by predominant currents (e.g. *Parablennius ruber* [77]).

### 4.3. Glacial refugia

For *L. bergylta*, the present results suggest three distinct refugia during glacial maxima: North Sea, western and southern Iberian coasts and Azores. Considering the fact that *L. bergylta* is able to breed at high latitudes in southern Norway [21], it is very likely that it was able to survive in European shores during the last glaciation. During the LGM, the North Sea surface was almost entirely frozen [2]. However, ballan wrasse populations may have survived in the vicinity of the North Sea and in more southern refugia. In fact, *L. bergylta* could have used unglaciated parts of these shores as glacial refugia, as previously suggested for other north Atlantic fish species (e.g. [14,16]). Our results agree with this hypothesis, namely the reported deep networks and haplotypes shared between northern and southern populations along the northeastern Atlantic.

Western and southern Iberian shores could have kept their populations and may have functioned as glacial refugia for this species. According to several authors, during the LGM the temperatures could have been kept within the thermal limits tolerated by *L. bergylta* (e.g. [2,5]). While analysing the post-glacial recolonization events in the marine coastal environment, one must also consider the close dependence between dispersal patterns and the ecology, life-history traits and behaviour of the various organisms [7]. The relatively long PLD reported for *L. bergylta* is probably related to the low degree of genetic differentiation found in this study along European coasts. A long PLD might also be associated with the species' ability to long-distance dispersal in a single or a few generations. This would explain the existence of shared haplotypes between southern and northern locations for both markers, thus diluting the source effect of their glacial refugia.

Another potential refugium where *L. bergylta* may have survived Pleistocene glaciations is the Azores. Water temperatures in this Macaronesian archipelago would have been suitable for this species during the LGM, as the drop in sea surface temperature was moderate [78]. The distinct nature of Azorean haplotypes and the absence of gene flow between Azorean and Continental European locations support this hypothesis. The existence of an Azorean glacial refugium has been reported in the literature for several fish species (e.g. *Raja clavata* [79], *C. galerita* [62]).

### 4.4. Final remarks on species cohesiveness, phenotype plasticity and implications to management

The ballan wrasse is the target of local fisheries across its entire distribution range and is a resource of paramount importance for local fisheries in northern Spain (e.g. [22,24]). It is currently being used as a cleaner species in northern Europe salmonid fish farms (e.g. [25]). Its absence from the archipelago of Madeira (the species was reported in [34]), if confirmed in the next few years, suggests that *L. bergylta* may be under important selective pressures. This is particularly relevant in its southern distribution limit. In the future, under a warming climate prospective, this could imply a northward regression of this species along its southern limit. Therefore, a thorough monitoring programme should be conducted both in Madeira (where it seems to be currently absent) and also along the Portuguese continental shore.

It is important that the results of this paper are taken into account together with the recent attention given to the wide phenotypic plasticity [80] and the systematic status of the ballan wrasse [22,27–32]. Although this study did not control for morphotypes, and this is still an ongoing subject of research, results obtained in [32] using the same genetic markers point to the conclusion that our results are not affected by it.

## 5. Conclusion

On the one hand, this study points towards the common origin of the historical population of *L. bergylta* with shared haplotypes between the Azores and mainland and exchanges of migrants in both directions (S7 dataset). On the other hand, the mitochondrial results reveal a complete isolation of the Azorean archipelago. The present-day pattern of oceanographic currents in the northeastern Atlantic is likely to maintain and/or accentuate the isolation and distinctiveness of this insular population of the ballan wrasse. This situation can possibly lead to a case of incipient speciation, although endemic fish species are rare in this archipelago (see [8]). This trend for the genetic isolation of the Azores can be occasionally reversed by severe or atypical weather events (e.g. [68]).

The relatively long PLD of the ballan wrasse and the absence of geographical barriers could lead us to infer unconstrained gene flow along European shores. However, the patterns found for this species reveal a population substructure, which can reflect both a recolonization from two distinct glacial refugia and the circulation pattern along the continental coast.

Commercial exploitation, together with the ongoing taxonomic discussion on distinct morphotypes, suggests that special attention should be given to this species in the near future. The genetic structure and phylogeographic pattern of *L. bergylta* still need further enlightenment and it would be interesting to complement this study with microsatellites and/or genomic tools, comparing specimens from both the plain and the spotted morphotypes along their entire distribution area.

## Supplementary Material

Appendix 1 - PCR details

## Supplementary Material

Figure S1 - Mismatch distributions

## Supplementary Material

Table S1 - specimens and locations

## Supplementary Material

Table S2 - Corrected average pairwise differences

## Data accessibility

DNA sequences are deposited in GenBank (accession nos. KU751889-KU752182).
