# Supplementary material for: Historical gene flow constraints in a northeastern Atlantic fish: phylogeography of the ballan wrasse Labrus bergylta across its distribution range
Source: R Soc Open Sci. 2017 Feb 15;4(2):160773. doi: 10.1098/rsos.160773 (PMC5367310; doi:10.1098/rsos.160773)
Supplement: Appendix 1 - PCR details [file rsos160773supp1.pdf]

Appendix II

Polymerase Chain Reactions (PCR) were performed in a 20µl total reaction volume with 10 µl of REDExtract-N-ampl PCR reaction mix (Sigma – Aldrich), 0.8µl of each primer (10µM), 4.4µl of Sigma-water, and 4µl of template DNA. The mitochondrial control region (CR) and the first intron of the nuclear S7 ribosomal protein gene (S7) were amplified using the following pairs of primers: L-pro1 (5′ ACTCTCACCCCTAGCTCCCAAAG 3′) and H-DL1 (5′ CTGAAGTAGGAACCAGATGCCAG 3′) [38] for the CR, and S7RPEX1F (5′-TGG CCT CTT CCT TGG CCG TC-3′) and S7RPEX2R (5′-AAC TCG TCT GGC TTT TCG CC-3′) [39]. An initial denaturation at 94◦C for 7′ was followed by 35/30 cycles (denaturation at 94◦C for 30/45′′, annealing at 55◦C for 30/45′′, and extension at 72◦C for 1′; values CR/S7, respectively) and a final extension at 72◦C for 7′ on a Bio-Rad Mycycler thermal cycler. The same primers were used for the sequencing reaction, and the PCR products were purified and sequenced in STABVIDA (http://www.stabvida.net/).
