# Supplementary material for: Historical gene flow constraints in a northeastern Atlantic fish: phylogeography of the ballan wrasse Labrus bergylta across its distribution range
Source: R Soc Open Sci. 2017 Feb 15;4(2):160773. doi: 10.1098/rsos.160773 (PMC5367310; doi:10.1098/rsos.160773)
Supplement: Figure S1 - Mismatch distributions [file rsos160773supp2.pdf]

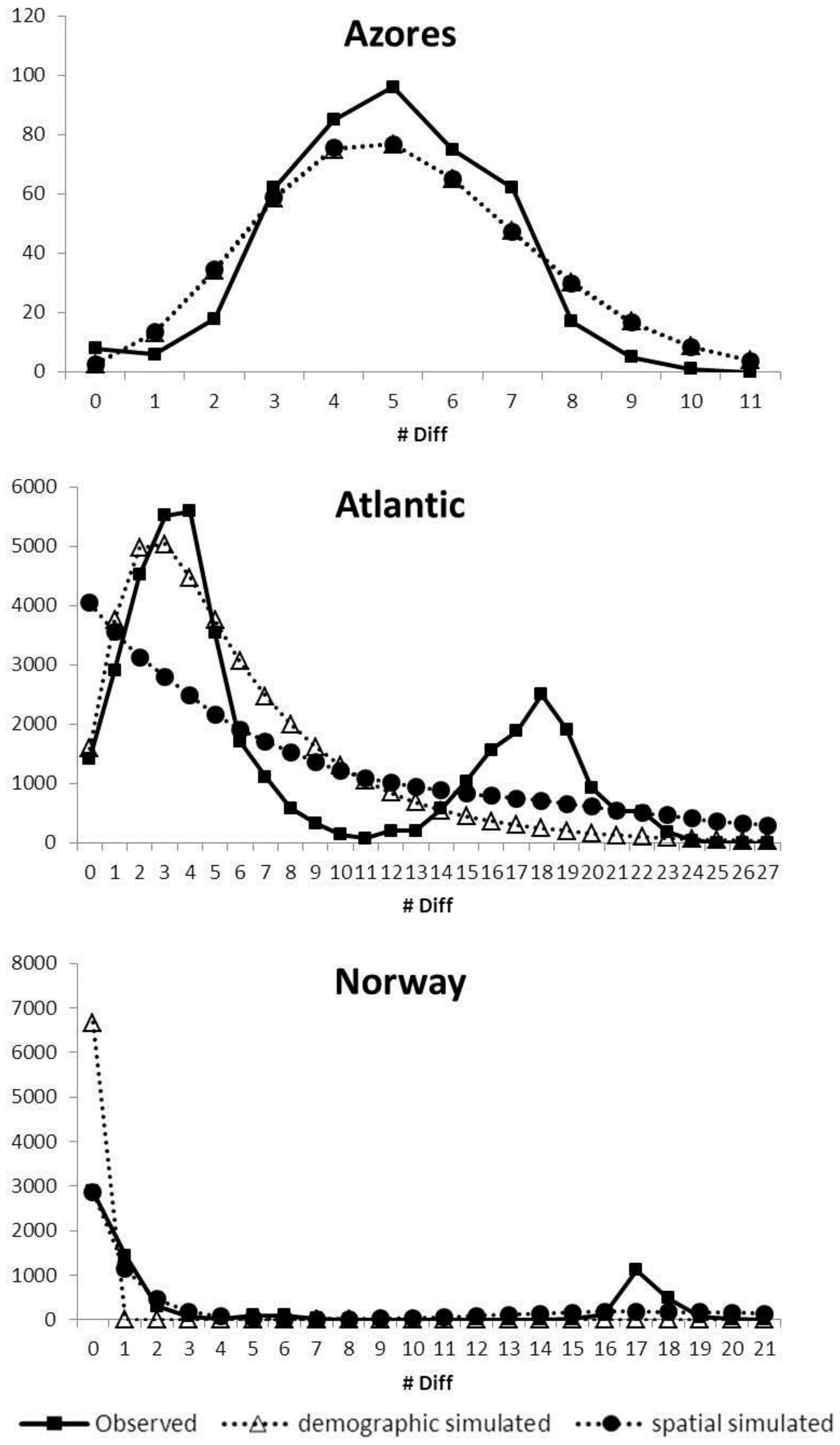

FigureS1 – Mismatch distributions for the CR of *Labrus bergylta*. Azores (Corvo and Santa Maria); Atlantic (Lisbon, Vigo, Ferrol, Roscoff, Portaferry, Mweenish, Bertraghboy Bay, Lochaline and Loch Sunart); Norway (Arendal, Hidra and Sogne).
