# Supplementary material for: Historical gene flow constraints in a northeastern Atlantic fish: phylogeography of the ballan wrasse Labrus bergylta across its distribution range
Source: R Soc Open Sci. 2017 Feb 15;4(2):160773. doi: 10.1098/rsos.160773 (PMC5367310; doi:10.1098/rsos.160773)
Supplement: Table S1 - specimens and locations [file rsos160773supp3.pdf]

Table S1. Sampling locations, specimens and GenBank Accession Numbers for CR and S7 of *Labrus bergylta*.

Sampling location	Specimen	CR			S7		
		Haplotype number	Accession number	Work	Haplotype number	Accession number	Work
Arendal	LBNWD1	-	-	-	47	KU306215	Almada et al., 2016
	LBNWD2	8	KU751890	present work	-	-	-
	LBNWP1	1	KU306119	Almada et al., 2016	-	-	-
	LBNWP2	52	KU306120	Almada et al., 2016	-	-	-
	LBNWP3	7	KU306121	Almada et al., 2016	-	-	-
	LBNWP4	10	KU306122	Almada et al., 2016	-	-	-
	LBNWP5	1	KU306123	Almada et al., 2016	-	-	-
	LBNWP6	1	KU306124	Almada et al., 2016	-	-	-
	LBNWS1	1	KU306162	Almada et al., 2016	-	-	-
	LBNWS2	1	KU306163	Almada et al., 2016	-	-	-
	LBNWS3	13	KU306164	Almada et al., 2016	-	-	-
	LBNWS4	1	KU306165	Almada et al., 2016	-	-	-
	LBNWS5	98	KU306166	Almada et al., 2016	-	-	-
	LBNWS6	98	KU306167	Almada et al., 2016	-	-	-
	LBNWS7	1	KU306168	Almada et al., 2016	-	-	-
	LBNWS8	7	KU306169	Almada et al., 2016	-	-	-
	LBNWS9	1	KU306170	Almada et al., 2016	-	-	-
	LBNWS10	1	KU306171	Almada et al., 2016	-	-	-
Hidra	NWH01	1	KC477846	D'Arcy et al., 2013	53	KU752104	present work
	NWH02	1	KC477846	D'Arcy et al., 2013	25	KU752105	present work
	NWH03	1	KC477846	D'Arcy et al., 2013	54	KU752106	present work
	NWH04	1	KC477846	D'Arcy et al., 2013	19	KU752107	present work
	NWH05	1	KC477846	D'Arcy et al., 2013	18	KU752108	present work
	NWH06	1	KC477846	D'Arcy et al., 2013	32	KU752109	present work
	NWH07	1	KC477846	D'Arcy et al., 2013	5	KU752110	present work
	NWH08	1	KC477846	D'Arcy et al., 2013	19	KU752111	present work
	NWH09	1	KC477846	D'Arcy et al., 2013	19	KU752112	present work
	NWH10	1	KC477846	D'Arcy et al., 2013	17	KU752113	present work
	NWH11	1	KC477846	D'Arcy et al., 2013	21	KU752114	present work
	NWH12	1	KC477846	D'Arcy et al., 2013	54	KU752115	present work
	NWH13	1	KC477846	D'Arcy et al., 2013	31	KU752116	present work
	NWH14	1	KC477846	D'Arcy et al., 2013	31	KU752117	present work
	NWH15	1	KC477846	D'Arcy et al., 2013	22	KU752118	present work
	NWH16	1	KC477846	D'Arcy et al., 2013	53	KU752119	present work
	NWH17	1	KC477846	D'Arcy et al., 2013	34	KU752120	present work
	NWH18	1	KC477846	D'Arcy et al., 2013	54	KU752121	present work
	NWH19	1	KC477846	D'Arcy et al., 2013	32	KU752122	present work
	NWH20	1	KC477846	D'Arcy et al., 2013	55	KU752123	present work
	NWH21	1	KC477846	D'Arcy et al., 2013	54	KU752124	present work
	NWH22	1	KC477846	D'Arcy et al., 2013	19	KU752125	present work
	NWH23	1	KC477846	D'Arcy et al., 2013	-	-	-
	NWH24	1	KC477846	D'Arcy et al., 2013	-	-	-
	NWH25	1	KC477846	D'Arcy et al., 2013	-	-	-
	NWH26	1	KC477846	D'Arcy et al., 2013	-	-	-
	NWH27	1	KC477846	D'Arcy et al., 2013	-	-	-

	NWH28	1	KC477846	D'Arcy et al., 2013	-	-	-
	NWH29	1	KC477846	D'Arcy et al., 2013	-	-	-
	NWH30	1	KC477846	D'Arcy et al., 2013	-	-	-
	NWH31	42	KC477847	D'Arcy et al., 2013	-	-	-
	NWH32	42	KC477847	D'Arcy et al., 2013	-	-	-
	NWH33	98	KC477850	D'Arcy et al., 2013	-	-	-
	NWH34	98	KC477850	D'Arcy et al., 2013	-	-	-
	NWH35	98	KC477850	D'Arcy et al., 2013	-	-	-
	NWH36	98	KC477850	D'Arcy et al., 2013	-	-	-
	NWH37	98	KC477850	D'Arcy et al., 2013	-	-	-
	NWH38	98	KC477850	D'Arcy et al., 2013	-	-	-
	NWH39	98	KC477850	D'Arcy et al., 2013	-	-	-
	NWH40	98	KC477850	D'Arcy et al., 2013	-	-	-
	NWH41	7	KC477859	D'Arcy et al., 2013	-	-	-
	NWH42	11	KC477860	D'Arcy et al., 2013	-	-	-
	NWH43	13	KC477869	D'Arcy et al., 2013	-	-	-
	NWH44	15	KC477871	D'Arcy et al., 2013	-	-	-
	NWH45	4	KC477922	D'Arcy et al., 2013	-	-	-
	NWH46	119	KC477923	D'Arcy et al., 2013	-	-	-
	NWH47	12	KC477924	D'Arcy et al., 2013	-	-	-
	NWH48	5	KC477925	D'Arcy et al., 2013	-	-	-
	NWH49	3	KC477926	D'Arcy et al., 2013	-	-	-
Sogne	NWS01	1	KC477846	D'Arcy et al., 2013	37	KU752126	present work
	NWS02	1	KC477846	D'Arcy et al., 2013	19	KU752128	present work
	NWS03	1	KC477846	D'Arcy et al., 2013	23	KU752129	present work
	NWS04	1	KC477846	D'Arcy et al., 2013	53	KU752130	present work
	NWS05	1	KC477846	D'Arcy et al., 2013	54	KU752131	present work
	NWS06	1	KC477846	D'Arcy et al., 2013	19	KU752132	present work
	NWS07	1	KC477846	D'Arcy et al., 2013	32	KU752133	present work
	NWS08	1	KC477846	D'Arcy et al., 2013	52	KU752134	present work
	NWS09	1	KC477846	D'Arcy et al., 2013	5	KU752135	present work
	NWS10	1	KC477846	D'Arcy et al., 2013	25	KU752136	present work
	NWS11	1	KC477846	D'Arcy et al., 2013	29	KU752137	present work
	NWS12	1	KC477846	D'Arcy et al., 2013	56	KU752138	present work
	NWS13	1	KC477846	D'Arcy et al., 2013	57	KU752139	present work
	NWS14	1	KC477846	D'Arcy et al., 2013	31	KU752140	present work
	NWS15	1	KC477846	D'Arcy et al., 2013	56	KU752141	present work
	NWS16	1	KC477846	D'Arcy et al., 2013	58	KU752142	present work
	NWS17	1	KC477846	D'Arcy et al., 2013	25	KU752143	present work
	NWS18	1	KC477846	D'Arcy et al., 2013	25	KU752144	present work
	NWS19	1	KC477846	D'Arcy et al., 2013	18	KU752145	present work
	NWS20	1	KC477846	D'Arcy et al., 2013	19	KU752146	present work
	NWS21	1	KC477846	D'Arcy et al., 2013	29	KU752147	present work
	NWS22	1	KC477846	D'Arcy et al., 2013	19	KU752148	present work
	NWS23	1	KC477846	D'Arcy et al., 2013	38	KU752127	present work
	NWS24	1	KC477846	D'Arcy et al., 2013	-	-	-
	NWS25	1	KC477846	D'Arcy et al., 2013	-	-	-
	NWS26	1	KC477846	D'Arcy et al., 2013	-	-	-
	NWS27	1	KC477846	D'Arcy et al., 2013	-	-	-
	NWS28	1	KC477846	D'Arcy et al., 2013	-	-	-
	NWS29	1	KC477846	D'Arcy et al., 2013	-	-	-

	NWS30	1	KC477846	D'Arcy et al., 2013	-	-	-
	NWS31	1	KC477846	D'Arcy et al., 2013	-	-	-
	NWS32	1	KC477846	D'Arcy et al., 2013	-	-	-
	NWS33	1	KC477846	D'Arcy et al., 2013	-	-	-
	NWS34	1	KC477846	D'Arcy et al., 2013	-	-	-
	NWS35	1	KC477846	D'Arcy et al., 2013	-	-	-
	NWS36	1	KC477846	D'Arcy et al., 2013	-	-	-
	NWS37	98	KC477850	D'Arcy et al., 2013	-	-	-
	NWS38	98	KC477850	D'Arcy et al., 2013	-	-	-
	NWS39	98	KC477850	D'Arcy et al., 2013	-	-	-
	NWS40	7	KC477859	D'Arcy et al., 2013	-	-	-
	NWS41	7	KC477859	D'Arcy et al., 2013	-	-	-
	NWS42	11	KC477860	D'Arcy et al., 2013	-	-	-
	NWS43	11	KC477860	D'Arcy et al., 2013	-	-	-
	NWS44	13	KC477869	D'Arcy et al., 2013	-	-	-
	NWS45	2	KC477870	D'Arcy et al., 2013	-	-	-
	NWS46	2	KC477870	D'Arcy et al., 2013	-	-	-
	NWS47	6	KC477918	D'Arcy et al., 2013	-	-	-
	NWS48	61	KC477919	D'Arcy et al., 2013	-	-	-
	NWS49	10	KC477920	D'Arcy et al., 2013	-	-	-
	NWS50	16	KC477921	D'Arcy et al., 2013	-	-	-
Loch Sunart	SCOTLS01	1	KC477846	D'Arcy et al., 2013	18	KU752173	present work
	SCOTLS02	1	KC477846	D'Arcy et al., 2013	-	-	-
	SCOTLS03	42	KC477847	D'Arcy et al., 2013	28	KU752174	present work
	SCOTLS04	42	KC477847	D'Arcy et al., 2013	67	KU752175	present work
	SCOTLS05	42	KC477847	D'Arcy et al., 2013	34	KU752176	present work
	SCOTLS06	42	KC477847	D'Arcy et al., 2013	33	KU752177	present work
	SCOTLS07	42	KC477847	D'Arcy et al., 2013	68	KU752178	present work
	SCOTLS08	42	KC477847	D'Arcy et al., 2013	-	-	-
	SCOTLS09	42	KC477847	D'Arcy et al., 2013	69	KU752179	present work
	SCOTLS10	68	KC477848	D'Arcy et al., 2013	-	-	-
	SCOTLS11	64	KC477849	D'Arcy et al., 2013	29	KU752180	present work
	SCOTLS12	107	KC477851	D'Arcy et al., 2013	33	KU752181	present work
	SCOTLS13	107	KC477851	D'Arcy et al., 2013	-	-	-
	SCOTLS14	107	KC477851	D'Arcy et al., 2013	-	-	-
	SCOTLS15	17	KC477853	D'Arcy et al., 2013	13	KU752182	present work
	SCOTLS16	87	KC477854	D'Arcy et al., 2013	-	-	-
	SCOTLS17	77	KC477855	D'Arcy et al., 2013	-	-	-
	SCOTLS18	23	KC477857	D'Arcy et al., 2013	-	-	-
	SCOTLS19	93	KC477858	D'Arcy et al., 2013	-	-	-
	SCOTLS20	51	KC477932	D'Arcy et al., 2013	-	-	-
	SCOTLS21	18	KC477933	D'Arcy et al., 2013	-	-	-
	SCOTLS22	39	KC477934	D'Arcy et al., 2013	-	-	-
	SCOTLS23	79	KC477935	D'Arcy et al., 2013	-	-	-
	SCOTLS24	49	KC477936	D'Arcy et al., 2013	-	-	-
Lochaline	SCOTLA01	1	KC477846	D'Arcy et al., 2013	-	-	-
	SCOTLA02	1	KC477846	D'Arcy et al., 2013	25	KU752153	present work
	SCOTLA03	42	KC477847	D'Arcy et al., 2013	5	KU752154	present work
	SCOTLA04	42	KC477847	D'Arcy et al., 2013	54	KU752155	present work
	SCOTLA05	42	KC477847	D'Arcy et al., 2013	29	KU752156	present work
	SCOTLA06	42	KC477847	D'Arcy et al., 2013	13	KU752157	present work

	SCOTLA07	42	KC477847	D'Arcy et al., 2013	29	KU752158	present work
	SCOTLA08	42	KC477847	D'Arcy et al., 2013	35	KU752159	present work
	SCOTLA09	68	KC477848	D'Arcy et al., 2013	63	KU752160	present work
	SCOTLA10	64	KC477849	D'Arcy et al., 2013	64	KU752161	present work
	SCOTLA11	64	KC477849	D'Arcy et al., 2013	18	KU752162	present work
	SCOTLA12	64	KC477849	D'Arcy et al., 2013	23	KU752163	present work
	SCOTLA13	99	KC477852	D'Arcy et al., 2013	65	KU752164	present work
	SCOTLA14	87	KC477854	D'Arcy et al., 2013	34	KU752165	present work
	SCOTLA15	30	KC477856	D'Arcy et al., 2013	29	KU752166	present work
	SCOTLA16	93	KC477858	D'Arcy et al., 2013	66	KU752167	present work
	SCOTLA17	45	KC477863	D'Arcy et al., 2013	25	KU752168	present work
	SCOTLA18	21	KC477865	D'Arcy et al., 2013	16	KU752169	present work
	SCOTLA19	92	KC477867	D'Arcy et al., 2013	-	-	-
	SCOTLA20	65	KC477868	D'Arcy et al., 2013	19	KU752170	present work
	SCOTLA21	15	KC477871	D'Arcy et al., 2013	60	KU752171	present work
	SCOTLA22	134	KC477927	D'Arcy et al., 2013	19	KU752172	present work
	SCOTLA23	78	KC477928	D'Arcy et al., 2013	-	-	-
	SCOTLA24	124	KC477929	D'Arcy et al., 2013	-	-	-
	SCOTLA25	124	KC477930	D'Arcy et al., 2013	-	-	-
	SCOTLA26	20	KC477931	D'Arcy et al., 2013	-	-	-
Portaferry	IRLP01	42	KC477847	D'Arcy et al., 2025	28	KU752014	present work
	IRLP02	42	KC477847	D'Arcy et al., 2026	29	KU752015	present work
	IRLP03	64	KC477849	D'Arcy et al., 2013	19	KU752016	present work
	IRLP04	107	KC477851	D'Arcy et al., 2013	29	KU752017	present work
	IRLP05	17	KC477853	D'Arcy et al., 2013	30	KU752018	present work
	IRLP06	31	KC477861	D'Arcy et al., 2013	22	KU752019	present work
	IRLP07	70	KC477910	D'Arcy et al., 2013	25	KU752020	present work
	IRLP08	128	KC477911	D'Arcy et al., 2013	19	KU752021	present work
	IRLP09	100	KC477912	D'Arcy et al., 2013	31	KU752022	present work
	IRLP10	122	KC477913	D'Arcy et al., 2013	5	KU752023	present work
	IRLP11	133	KC477914	D'Arcy et al., 2013	5	KU752024	present work
	IRLP12	69	KC477915	D'Arcy et al., 2013	28	KU752025	present work
	IRLP13	48	KC477916	D'Arcy et al., 2013	-	-	-
	IRLP14	27	KC477917	D'Arcy et al., 2013	-	-	-
Bertraghboy	IRLB01	1	KC477846	D'Arcy et al., 2013	-	-	-
	IRLB02	42	KC477847	D'Arcy et al., 2013	-	-	-
	IRLB03	42	KC477847	D'Arcy et al., 2013	-	-	-
	IRLB04	42	KC477847	D'Arcy et al., 2013	-	-	-
	IRLB05	42	KC477847	D'Arcy et al., 2013	-	-	-
	IRLB06	42	KC477847	D'Arcy et al., 2013	-	-	-
	IRLB07	42	KC477847	D'Arcy et al., 2013	-	-	-
	IRLB08	42	KC477847	D'Arcy et al., 2013	-	-	-
	IRLB09	68	KC477848	D'Arcy et al., 2013	13	KU751990	present work
	IRLB10	68	KC477848	D'Arcy et al., 2013	-	-	-
	IRLB11	68	KC477848	D'Arcy et al., 2013	14	KU751991	present work
	IRLB12	64	KC477849	D'Arcy et al., 2013	-	-	-
	IRLB13	64	KC477849	D'Arcy et al., 2013	-	-	-
	IRLB14	107	KC477851	D'Arcy et al., 2013	-	-	-
	IRLB15	107	KC477851	D'Arcy et al., 2013	-	-	-
	IRLB16	99	KC477852	D'Arcy et al., 2013	-	-	-
	IRLB17	17	KC477853	D'Arcy et al., 2013	-	-	-

	IRLB18	17	KC477853	D'Arcy et al., 2013	-	-	-
	IRLB19	87	KC477854	D'Arcy et al., 2013	-	-	-
	IRLB20	77	KC477855	D'Arcy et al., 2013	-	-	-
	IRLB21	30	KC477856	D'Arcy et al., 2013	-	-	-
	IRLB22	23	KC477857	D'Arcy et al., 2013	-	-	-
	IRLB23	23	KC477857	D'Arcy et al., 2013	-	-	-
	IRLB24	93	KC477858	D'Arcy et al., 2013	-	-	-
	IRLB25	120	KC477864	D'Arcy et al., 2013	-	-	-
	IRLB26	44	KC477866	D'Arcy et al., 2013	-	-	-
	IRLB27	92	KC477867	D'Arcy et al., 2013	-	-	-
	IRLB28	65	KC477868	D'Arcy et al., 2013	-	-	-
	IRLB29	56	KC477894	D'Arcy et al., 2013	-	-	-
	IRLB30	123	KC477895	D'Arcy et al., 2013	-	-	-
	IRLB31	24	KC477896	D'Arcy et al., 2013	-	-	-
	IRLB32	32	KC477897	D'Arcy et al., 2013	-	-	-
	IRLB33	95	KC477898	D'Arcy et al., 2013	-	-	-
	IRLB34	125	KC477899	D'Arcy et al., 2013	-	-	-
	IRLB35	41	KC477900	D'Arcy et al., 2013	-	-	-
	IRLB36	40	KC477901	D'Arcy et al., 2013	-	-	-
	IRLB37	9	KC477902	D'Arcy et al., 2013	-	-	-
	IRLB38	33	KC477903	D'Arcy et al., 2013	-	-	-
	IRLB39	127	KC477904	D'Arcy et al., 2013	-	-	-
	IRLB40	126	KC477905	D'Arcy et al., 2013	-	-	-
	IRLB41	101	KC477906	D'Arcy et al., 2013	-	-	-
	IRLB42	59	KC477907	D'Arcy et al., 2013	-	-	-
	IRLB43	109	KC477908	D'Arcy et al., 2013	-	-	-
	IRLB44	61	KC477909	D'Arcy et al., 2013	-	-	-
Mweenish	IRLM01	1	KC477846	D'Arcy et al., 2013	15	KU751992	present work
	IRLM02	42	KC477847	D'Arcy et al., 2013	16	KU751993	present work
	IRLM03	42	KC477847	D'Arcy et al., 2013	17	KU751994	present work
	IRLM04	42	KC477847	D'Arcy et al., 2013	18	KU751995	present work
	IRLM05	42	KC477847	D'Arcy et al., 2013	19	KU751996	present work
	IRLM06	42	KC477847	D'Arcy et al., 2013	20	KU751997	present work
	IRLM07	42	KC477847	D'Arcy et al., 2013	21	KU751998	present work
	IRLM08	68	KC477848	D'Arcy et al., 2013	22	KU751999	present work
	IRLM09	68	KC477848	D'Arcy et al., 2013	23	KU752000	present work
	IRLM10	68	KC477848	D'Arcy et al., 2013	22	KU752001	present work
	IRLM11	68	KC477848	D'Arcy et al., 2013	5	KU752002	present work
	IRLM12	68	KC477848	D'Arcy et al., 2013	24	KU752003	present work
	IRLM13	68	KC477848	D'Arcy et al., 2013	25	KU752004	present work
	IRLM14	68	KC477848	D'Arcy et al., 2013	25	KU752005	present work
	IRLM15	68	KC477848	D'Arcy et al., 2013	26	KU752006	present work
	IRLM16	68	KC477848	D'Arcy et al., 2013	18	KU752007	present work
	IRLM17	64	KC477849	D'Arcy et al., 2013	27	KU752008	present work
	IRLM18	64	KC477849	D'Arcy et al., 2013	22	KU752009	present work
	IRLM19	64	KC477849	D'Arcy et al., 2013	18	KU752010	present work
	IRLM20	64	KC477849	D'Arcy et al., 2013	19	KU752011	present work
	IRLM21	107	KC477851	D'Arcy et al., 2013	28	KU752012	present work
	IRLM22	107	KC477851	D'Arcy et al., 2013	18	KU752013	present work
	IRLM23	107	KC477851	D'Arcy et al., 2013	-	-	-
	IRLM24	107	KC477851	D'Arcy et al., 2013	-	-	-

	IRLM25	99	KC477852	D'Arcy et al., 2013	-	-	-
	IRLM26	99	KC477852	D'Arcy et al., 2013	-	-	-
	IRLM27	99	KC477852	D'Arcy et al., 2013	-	-	-
	IRLM28	99	KC477852	D'Arcy et al., 2013	-	-	-
	IRLM29	99	KC477852	D'Arcy et al., 2013	-	-	-
	IRLM30	99	KC477852	D'Arcy et al., 2013	-	-	-
	IRLM31	17	KC477853	D'Arcy et al., 2013	-	-	-
	IRLM32	17	KC477853	D'Arcy et al., 2013	-	-	-
	IRLM33	17	KC477853	D'Arcy et al., 2013	-	-	-
	IRLM34	17	KC477853	D'Arcy et al., 2013	-	-	-
	IRLM35	87	KC477854	D'Arcy et al., 2013	-	-	-
	IRLM36	87	KC477854	D'Arcy et al., 2013	-	-	-
	IRLM37	87	KC477854	D'Arcy et al., 2013	-	-	-
	IRLM38	77	KC477855	D'Arcy et al., 2013	-	-	-
	IRLM39	77	KC477855	D'Arcy et al., 2013	-	-	-
	IRLM40	77	KC477855	D'Arcy et al., 2013	-	-	-
	IRLM41	30	KC477856	D'Arcy et al., 2013	-	-	-
	IRLM42	30	KC477856	D'Arcy et al., 2013	-	-	-
	IRLM43	23	KC477857	D'Arcy et al., 2013	-	-	-
	IRLM44	31	KC477861	D'Arcy et al., 2013	-	-	-
	IRLM45	83	KC477862	D'Arcy et al., 2013	-	-	-
	IRLM46	83	KC477862	D'Arcy et al., 2013	-	-	-
	IRLM47	45	KC477863	D'Arcy et al., 2013	-	-	-
	IRLM48	121	KC477864	D'Arcy et al., 2013	-	-	-
	IRLM49	21	KC477865	D'Arcy et al., 2013	-	-	-
	IRLM50	44	KC477866	D'Arcy et al., 2013	-	-	-
	IRLM51	19	KC477872	D'Arcy et al., 2013	-	-	-
	IRLM52	113	KC477873	D'Arcy et al., 2013	-	-	-
	IRLM53	89	KC477874	D'Arcy et al., 2013	-	-	-
	IRLM54	14	KC477875	D'Arcy et al., 2013	-	-	-
	IRLM55	43	KC477876	D'Arcy et al., 2013	-	-	-
	IRLM56	94	KC477877	D'Arcy et al., 2013	-	-	-
	IRLM57	131	KC477878	D'Arcy et al., 2013	-	-	-
	IRLM58	108	KC477879	D'Arcy et al., 2013	-	-	-
	IRLM59	36	KC477880	D'Arcy et al., 2013	-	-	-
	IRLM60	47	KC477881	D'Arcy et al., 2013	-	-	-
	IRLM61	106	KC477882	D'Arcy et al., 2013	-	-	-
	IRLM62	82	KC477883	D'Arcy et al., 2013	-	-	-
	IRLM63	85	KC477884	D'Arcy et al., 2013	-	-	-
	IRLM64	57	KC477885	D'Arcy et al., 2013	-	-	-
	IRLM65	84	KC477886	D'Arcy et al., 2013	-	-	-
	IRLM66	86	KC477887	D'Arcy et al., 2013	-	-	-
	IRLM67	46	KC477888	D'Arcy et al., 2013	-	-	-
	IRLM68	132	KC477889	D'Arcy et al., 2013	-	-	-
	IRLM69	38	KC477890	D'Arcy et al., 2013	-	-	-
	IRLM70	34	KC477891	D'Arcy et al., 2013	-	-	-
	IRLM71	28	KC477892	D'Arcy et al., 2013	-	-	-
	IRLM72	26	KC477893	D'Arcy et al., 2013	-	-	-
English Channel	CM1	87	KU751928	present work	29	KU752032	present work
Roscoff	RO01	68	KU751924	present work	17	KU752053	present work
	RO02	61	KU751909	present work	12	KU752054	present work

	RO03	72	KU751918	present work	-	-	-
	RO04	116	KU751945	present work	59	KU752149	present work
	RO05	58	KU751904	present work	-	-	-
	RO06	118	KU751947	present work	12	KU752055	present work
	RO07	73	KU751919	present work	32	KU752056	present work
	RO08	1	KU751889	present work	37	KU752057	present work
	RO09	42	KU751898	present work	32	KU752058	present work
	RO10	88	KU751930	present work	60	KU752150	present work
	RO11	47	KU751901	present work	-	-	-
	RO12	33	KU751892	present work	61	KU752151	present work
	RO13	76	KU751926	present work	13	KU752059	present work
	RO14	74	KU751920	present work	32	KU752060	present work
	RO15	63	KU751910	present work	12	KU752061	present work
	RO16	111	KU751940	present work	62	KU752152	present work
	RO17	129	KU751949	present work	-	-	-
	RO19	56	KU751906	present work	29	KU752062	present work
	RO20	42	KU751897	present work	13	KU752063	present work
	RO21	56	KU751907	present work	32	KU752064	present work
	RO23	42	KU751896	present work	5	KU752065	present work
	RO24	103	KU751936	present work	35	KU752066	present work
	RO25	22	KU751891	present work	-	-	-
	RO26	60	KU751908	present work	33	KU752067	present work
	RO27	42	KU751895	present work	38	KU752068	present work
	RO28	35	KU751893	present work	28	KU752069	present work
	RO29	58	KU751905	present work	32	KU752070	present work
	RO30	-	-	-	35	KU752071	present work
Ferrol	GAL1	67	KU306172	Almada et al., 2016	5	KU306220	Almada et al., 2016
	GAL2	119	KU306173	Almada et al., 2016	5	KU306221	Almada et al., 2016
	GAL3	64	KU306125	Almada et al., 2016	13	KU306187	Almada et al., 2016
	GAL4	64	KU751911	present work	32	KU752043	present work
	GAL5	49	KU306126	Almada et al., 2016	32	KU306188	Almada et al., 2016
	GAL6	92	KU306127	Almada et al., 2016	16	KU306189	Almada et al., 2016
	GAL7	56	KU306174	Almada et al., 2016	5	KU306222	Almada et al., 2016
	GAL8	102	KX663834	present work	18	KU752044	present work
	GAL9	89	KU306128	Almada et al., 2016	5	KU306190	Almada et al., 2016
	GAL10	114	KU751943	present work	35	KU752033	present work
	GAL11	92	KU306129	Almada et al., 2016	-	-	-
	GAL12	42	KU306130	Almada et al., 2016	33	KU306191	Almada et al., 2016
	GAL13	68	KU751921	present work	33	KU752034	present work
	GAL14	112	KU751942	present work	33	KU752035	present work
	GAL16	68	KU306131	Almada et al., 2016	32	KU306192	Almada et al., 2016
	GAL17	56	KU306175	Almada et al., 2016	-	-	-
	GAL18	64	KU751915	present work	35	KU752036	present work
	GAL19	135	KU306132	Almada et al., 2016	33	KU306193	Almada et al., 2016
	GAL20	71	KU751917	present work	17	KU752037	present work
	GAL21	64	KU751912	present work	6	KU751985	present work
Vigo	GAL22	37	KU751894	present work	29	KU752038	present work
	GAL23	119	KU751948	present work	5	KU752039	present work
	GAL24	65	KU751913	present work	34	KU752040	present work
	GAL25	96	KU751933	present work	7	KU751986	present work
	GAL26	87	KU751929	present work	8	KU751987	present work

	GAL27	92	KU751935	present work	33	KU752041	present work
	GAL28	104	KU751937	present work	35	KU752042	present work
	GAL29	110	KU751939	present work	9	KU751988	present work
	GAL30	115	KU751944	present work	10	KU751989	present work
	GAL52	92	KU306176	Almada et al., 2016	11	KU306223	Almada et al., 2016
	GAL53	68	KU306177	Almada et al., 2016	-	-	-
	GAL54	54	KU306178	Almada et al., 2016	32	KU306224	Almada et al., 2016
	GAL55	50	KU306179	Almada et al., 2016	12	KU306225	Almada et al., 2016
	GAL56	55	KU306180	Almada et al., 2016	18	KU306226	Almada et al., 2016
	GAL57	92	KU306181	Almada et al., 2016	-	-	-
	GAL58	33	KU306133	Almada et al., 2016	-	-	-
	GAL59	99	KU306134	Almada et al., 2016	-	-	-
	GAL60	99	KU306135	Almada et al., 2016	-	-	-
	GAL61	64	KU306136	Almada et al., 2016	-	-	-
	GAL62	77	KU306137	Almada et al., 2016	-	-	-
	GAL63	64	KU306138	Almada et al., 2016	-	-	-
Lisbon	LIS1	61	KU306139	Almada et al., 2016	32	KU306195	Almada et al., 2016
	LIS2	105	KU306140	Almada et al., 2016	29	KU306196	Almada et al., 2016
	LIS3	42	KU751900	present work	10	KU752051	present work
	LIS4	107	KU306141	Almada et al., 2016	10	KU306197	Almada et al., 2016
	LIS5	81	KU306142	Almada et al., 2016	29	KU306198	Almada et al., 2016
	LIS6	17	KU306143	Almada et al., 2016	36	KU306199	Almada et al., 2016
	LIS7	117	KU306144	Almada et al., 2016	52	KU306200	Almada et al., 2016
	LIS8	99	KU751938	present work	36	KU306227	Almada et al., 2016
	LIS9	42	KU306145	Almada et al., 2016	32	KU306201	Almada et al., 2016
	LIS10	90	KU306146	Almada et al., 2016	23	KU306202	Almada et al., 2016
	LIS11	62	KU306147	Almada et al., 2016	32	KU306203	Almada et al., 2016
	LIS12	80	KU306148	Almada et al., 2016	32	KU306204	Almada et al., 2016
	LIS13	77	KU306183	Almada et al., 2016	49	KU306228	Almada et al., 2016
	LIS14	99	KU306149	Almada et al., 2016	32	KU306205	Almada et al., 2016
	LIS15	25	KU306150	Almada et al., 2016	5	KU306206	Almada et al., 2016
	LIS16	92	KU306151	Almada et al., 2016	13	KU306207	Almada et al., 2016
	LIS17	29	KU306152	Almada et al., 2016	18	KU306208	Almada et al., 2016
	LIS18	107	KU306153	Almada et al., 2016	5	KU306209	Almada et al., 2016
	LIS19	77	KU306154	Almada et al., 2016	36	KU306210	Almada et al., 2016
	LIS20	53	KU306155	Almada et al., 2016	13	KU306211	Almada et al., 2016
	LIS21	99	KU306156	Almada et al., 2016	50	KU306212	Almada et al., 2016
	LIS22	114	KU751946	present work	27	KU752102	present work
	LIS23	75	KU751925	present work	17	KU752045	present work
	LIS24	107	KU751941	present work	32	KU752046	present work
	LIS25	130	KU751950	present work	32	KU752047	present work
	LIS26	68	KU751922	present work	29	KU752048	present work
	LIS27	68	KU751923	present work	23	KU752049	present work
	LIS28	97	KU751934	present work	29	KU752050	present work
	LIS29	42	KU751902	present work	51	KU752103	present work
	LIS30	42	KU751899	present work	33	KU752052	present work
	ARR01	42	KU306157	Almada et al., 2016	32	KU306213	Almada et al., 2016
	ARR2	42	KU751903	present work	33	KU752030	present work
	ARR3	68	KU751916	present work	4	KU751984	present work
	ARR4	66	KU751914	present work	34	KU752031	present work
Martinhal	ALG1	42	KU306158	Almada et al., 2016	32	KU752027	present work

	ALG2	121	KU306159	Almada et al., 2016	5	KU752028	present work
	ALG3	68	KU306160	Almada et al., 2016	32	KU752029	present work
Cadiz	LBSP01	-	-	-	48	KU752099	present work
	LBSP02	92	KU751932	present work	32	KU752100	present work
	LBSP03	91	KU751931	present work	32	KU752101	present work
Canary Islands	LBCAN01	77	KU751927	present work	-	-	-
Corvo	LB01A	159	KU751980	present work	-	-	-
	LB01B	155	KU751974	present work	40	KU752084	present work
	LB02A	152	KU751971	present work	-	-	-
	LB03A	144	KU751960	present work	29	KU752074	present work
	LB04A	158	KU751979	present work	41	KU752095	present work
	LB4A	-	-	-	-	-	-
	LB13	138	KU751953	present work	29	KU752079	present work
	LB15	143	KU751959	present work	41	KU752080	present work
	LB17	150	KU751969	present work	41	KU752083	present work
	LB30	156	KU751976	present work	42	KU752086	present work
	LB31	142	KU751957	present work	40	KU752087	present work
	LB33	142	KU751958	present work	44	KU752089	present work
	LB34	151	KU751970	present work	41	KU752090	present work
	LB35	156	KU751977	present work	41	KU752091	present work
	LB36	154	KU751973	present work	45	KU752092	present work
	LB37	144	KU751961	present work	-	-	-
Faial	AC1	136	KU306161	Almada et al., 2016	1	KU306214	Almada et al., 2016
	AC2	144	KU306184	Almada et al., 2016	2	KU306229	Almada et al., 2016
	AC3	147	KU751963	present work	3	KU751983	present work
	AC4	136	KU751951	present work	29	KU752026	present work
	AC5	145	KU306185	Almada et al., 2016	29	KU306230	Almada et al., 2016
	LBERAC1	-	-	-	46	KU752097	present work
	LBERAC2	-	-	-	41	KU752098	present work
Stª Maria	LB01	148	KU751965	present work	39	KU752072	present work
	LB02	140	KU751955	present work	29	KU752073	present work
	LB03	153	KU751972	present work	-	-	-
	LB04	149	KU751966	present work	41	KU752094	present work
	LB06	146	KU751962	present work	40	KU752075	present work
	LB10	139	KU751954	present work	41	KU752076	present work
	LB11	156	KU751975	present work	40	KU752077	present work
	LB12	137	KU751952	present work	41	KU752078	present work
	LB15A	160	KU751981	present work	41	KU752081	present work
	LB16	147	KU751964	present work	42	KU752082	present work
	LB29	141	KU751956	present work	41	KU752085	present work
	LB30A	161	KU751982	present work	-	-	-
	LB32	149	KU751967	present work	43	KU752088	present work
	LB37A	157	KU751978	present work	41	KU752093	present work
	LB54	149	KU751968	present work	41	KU752096	present work
