# Supplementary material for: Historical gene flow constraints in a northeastern Atlantic fish: phylogeography of the ballan wrasse Labrus bergylta across its distribution range
Source: R Soc Open Sci. 2017 Feb 15;4(2):160773. doi: 10.1098/rsos.160773 (PMC5367310; doi:10.1098/rsos.160773)
Supplement: Table S2 - Corrected average pairwise differences [file rsos160773supp4.pdf]

Table S2. Corrected average pairwise differences among collecting sites of *Labrus bergylta*: CR (above diagonal) and S7 (below diagonal). Significant values of probability *P* are shown in bold.

	AR	HI	SO	LS	LO	PO	BB	ME	RO	FE	VI	LI	SM	CO
AR		-0.150	-0.026	**6.362**	**6.268**	**6.416**	**6.012**	**6.575**	**7.925**	**10.478**	**8.343**	**8.867**	**20.114**	**19.662**
HI	-		**0.253**	**5.350**	**5.256**	**5.395**	**5.025**	**5.547**	**6.818**	**9.178**	**7.155**	**7.672**	**19.876**	**19.334**
SO	-	-0.020		**8.336**	**8.257**	**8.408**	**7.929**	**8.552**	**10.113**	**12.993**	**10.601**	**11.174**	**21.618**	**21.129**
LS	-	**0.104**	0.094		-0.272	-0.328	-0.182	-0.128	-0.042	**0.584**	0.112	0.098	**19.300**	**18.596**
LO	-	0.019	0.026	-0.015		-0.333	-0.148	-0.117	-0.021	**0.505**	0.031	0.100	**19.251**	**18.566**
PO	-	-0.004	-0.020	0.076	0.037		-0.319	-0.287	-0.110	**0.438**	-0.082	-0.025	**19.040**	**18.411**
BB	-	-	-	-	-	-		-0.100	0.149	**0.823**	0.193	0.278	**19.102**	**18.430**
ME	-	0.030	0.021	-0.001	0.003	-0.005	-		0.100	**0.650**	0.100	0.170	**19.468**	**18.828**
RO	-	**0.117**	**0.117**	0.069	**0.061**	**0.190**	-	**0.102**		0.140	-0.021	-0.072	**19.460**	**18.849**
FE	-	**0.085**	**0.093**	-0.007	0.014	**0.128**	-	**0.062**	0.005		0.088	0.040	**21.493**	**20.836**
VI	-	**0.070**	**0.080**	-0.060	-0.024	0.060	-	0.001	**0.096**	0.009		-0.072	**20.807**	**20.098**
LI	-	**0.102**	**0.123**	0.057	0.029	**0.179**	-	**0.091**	0.004	0.008	**0.065**		**20.522**	**19.871**
SM	-	**0.828**	**0.820**	**0.498**	**0.621**	**0.824**	-	**0.628**	**0.527**	**0.510**	**0.585**	**0.493**		0.054
CO	-	**0.736**	**0.728**	**0.406**	**0.528**	**0.732**	-	**0.536**	**0.435**	**0.418**	**0.492**	**0.401**	-0.006	
